# Supplementary material for: Aggregating residue-level protein language model embeddings with optimal transport
Source: Bioinform Adv. 2025 Mar 20;5(1):vbaf060. doi: 10.1093/bioadv/vbaf060 (PMC11961220; doi:10.1093/bioadv/vbaf060)
Supplement: vbaf060_Supplementary_Data [file vbaf060_supplementary_data.pdf]

Aggregating Residue-Level Protein Language Model Embeddings with Optimal Transport: Supplementary Material

Navid NaderiAlizadeh and Rohit Singh

A Training Settings and Hyperparameters

For the DTI and SCL tasks, we train the SWE aggregation parameters using the AdamW optimizer [S5] with a learning rate of 10^{-4} and cosine annealing schedule with a restart duration of 10 epochs. For the EC task, we train the SWE aggregation parameters using the Adam optimizer [S4] with a learning rate of 10^{-3}, reducing it by a factor of 0.6 if the performance does not improve after 5 epochs. The batch size is set to 32 for the DTI and SCL tasks and 8 for the EC task.

B Binary DTI Prediction AUROC Results

Figure S1 shows the AUROC comparison between the proposed pooling method and the baselines for the DTI prediction task on DAVIS and Binding-DB for ESM-2 and ProGen2 models. The overall trends and gains of SWE with respect to baseline pooling operations are consistent with our AUPR results in Figure 2.

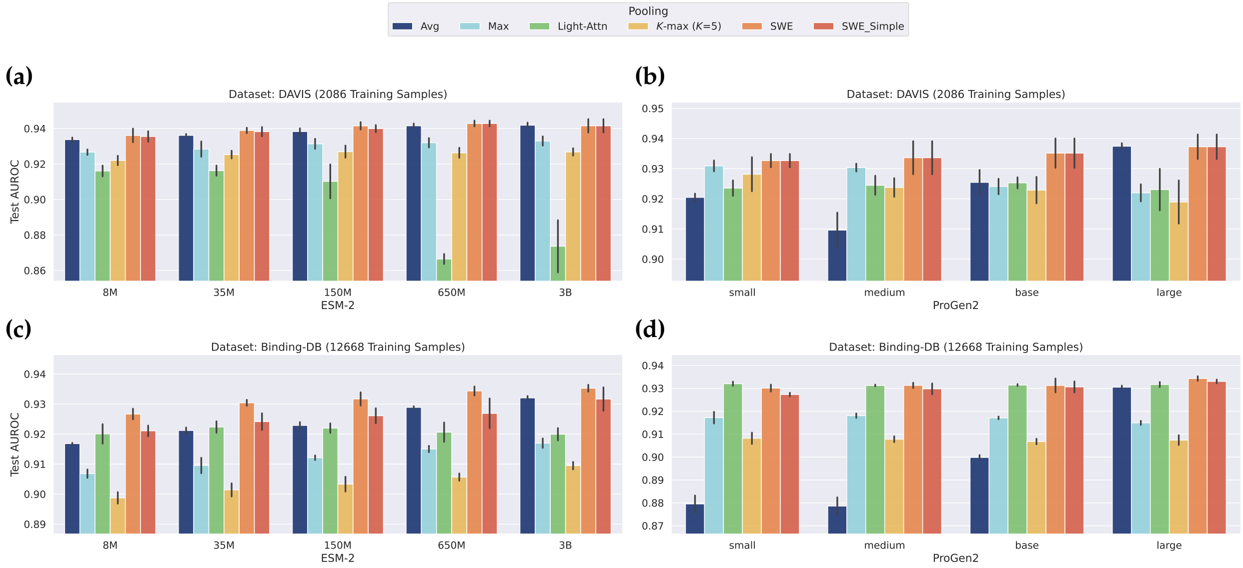

Figure S1: Test AUROC performance of SWE and SWE_Simple as compared to baseline pooling methods for the binary drug-target interaction task on the DAVIS (top) and Binding-DB (bottom) datasets. Performance is evaluated with two families of PLMs, namely ESM-2 (left) and ProGen2 (right).

Moreover, Figure S2 shows the AUROC gains of SWE over average pooling across different protein lengths. Similar to the AUPR gains in Figure 3, SWE's gains over average pooling increase with the sequence length and decrease with the PLM size.

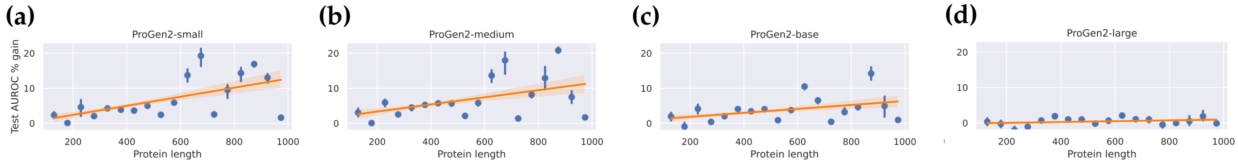

Figure S2: Test AUROC gains of SWE over average pooling vs. the lengths of the proteins' amino acid sequences across four ProGen2 PLMs on Binding-DB.

C Comparison with [CLS] Embeddings

Figure S3 shows the performance comparison between average pooling, SWE, and the [CLS] token embedding for the DTI prediction tasks with ESM-2 PLMs. As the figure demonstrates, average pooling and [CLS] embedding present complementary strengths, with [CLS] outperforming average pooling with larger PLMs on DAVIS and the smallest PLM on Binding-DB (in terms of test AUPR). The proposed SWE method exhibits robust performance, outperforming both methods across all scenarios.

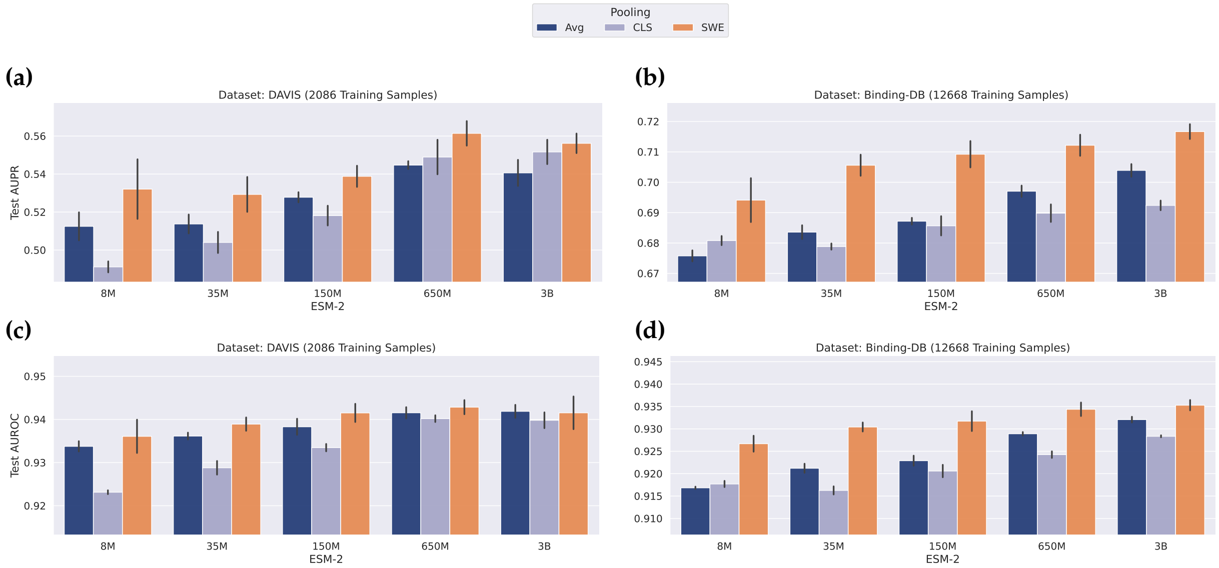

Figure S3: Test AUPR (top) and AUROC (bottom) performance of SWE as compared to average pooling and [CLS] embedding for the binary drug-target interaction task on the DAVIS (left) and Binding-DB (right) datasets. Performance is evaluated with ESM-2 PLMs.

D SWE Validation Results

As mentioned in the paper, we perform a hyperparameter grid search for SWE, where we find the best combination of $m \in \{100, \ldots, 1000\}$, as well as whether or not to freeze the reference and slicer parameters, which yields the best *validation* performance for any given task. Figure S4 illustrates the validation performance of the 20 aforementioned SWE configurations across the DTI, SCL, and EC tasks. As the figure shows, the optimal configuration depends on the task at hand. In case such an extensive hyperparameter grid search is not feasible, we recommend setting $m = 1000$ and only performing a grid search on the binary "freeze" hyperparameter.

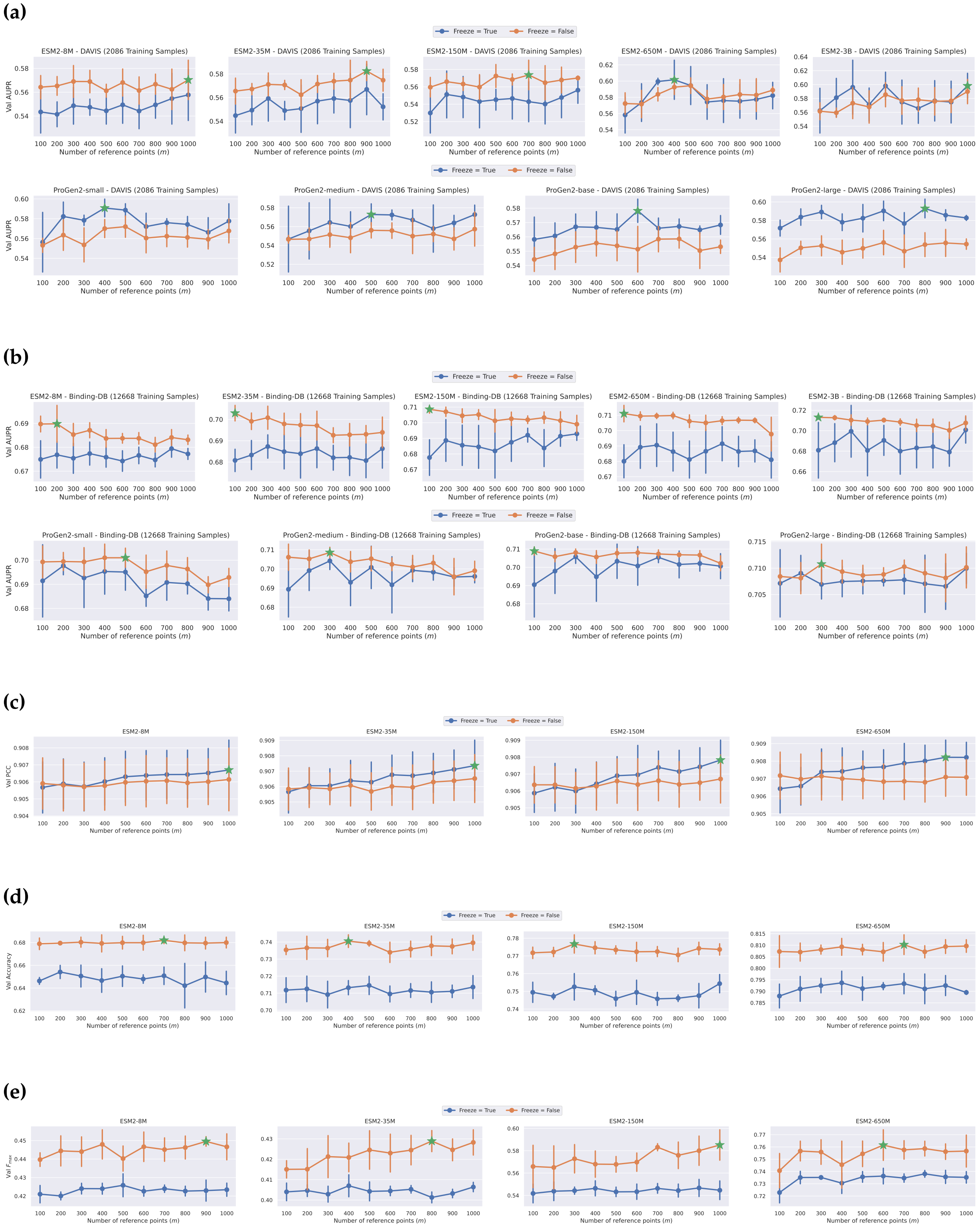

Figure S4: Validation performance of SWE with different (m, freeze) pair configurations in the **(a)** DAVIS drug-target interaction prediction, **(b)** Binding-DB drug-target interaction prediction, **(c)** drug-target affinity prediction, **(d)** subcellular localization, and **(e)** EC prediction tasks. The green stars mark the optimal configurations used for testing.

E Protein-Protein Interaction (PPI) Prediction

In the third task, we focus on predicting whether or not two given proteins will interact with each other. Specifically, we embed each protein's amino acid sequence using the same PLM and aggregation pipeline in parallel, and then we leverage the cosine similarity between the protein-level representations to estimate their interaction probability. For a given PPI training dataset consisting of N (protein, protein, label) triplets $\{(\overline{\mathbf{p}_j}, \underline{\mathbf{p}_j}, y_j)\}_{j=1}^{N}$, this task seeks to solve the following supervised learning problem:

$$\min_{\theta_\pi \in \Theta_\pi, \mathbf{V} \in \mathbb{R}^{D \times d}} \frac{1}{N} \sum_{j=1}^{N} \ell_{\mathsf{CosBCE}} \left(\sigma \left(\mathbf{V} \psi(\overline{\mathbf{p}_j}; \theta_\phi, \theta_\pi) \right), \sigma \left(\mathbf{V} \psi(\underline{\mathbf{p}_j}; \theta_\phi, \theta_\pi) \right), y_j \right).$$

We use the "gold standard" dataset provided by [S3] to evaluate our proposed SWE aggregation mechanism in this task, which comprises balanced PPI data without any leakage between training, validation, and testing samples. For this specific task, we consider four options for the number of slices, $L \in \{128, 256, 512, 1024\}$, as well as four options for the number of reference points, $m \in \{128, 256, 512, 1024\}$, leading to 16 different SWE configurations. While the default batch size is 32, for experiments with $L = 1024$ slices, we use a reduced batch size of 24 due to computational limitations. We report the mean and standard deviation of the test/validation performance for the SWE configuration (i.e., (L, m) pair) with the highest validation AUPR across the 50 training epochs.

Table S1 compares the validation and test performance of our proposed SWE aggregation method with average pooling across the considered ESM-2 PLMs. As the table shows, SWE generally outperforms average pooling in terms of F1-score and recall, while performing on par with average pooling in terms of accuracy, and underperforming for the other metrics, including precision and specificity. These results are consistent with the ones reported by [S7], where more expressive models and heavier fine-tuning lead to superior F1 and recall levels, while lowering the other metrics. Further research, including using optimal partial transport- (OPT-)based embedding methods [S1, S2], is required to strike the right balance among the different metrics through which PPI prediction quality is measured.

	ESM-2 PLM	Aggregation	Accuracy	F1	MCC	AUPR	Precision	Recall	Specificity
Validation	8M	SWE	0.607 ± 0.003	0.626 ± 0.008	0.216 ± 0.006	0.652 ± 0.004	0.619 ± 0.006	0.671 ± 0.023	0.701 ± 0.013
		Avg	0.606 ± 0.002	0.614 ± 0.004	0.216 ± 0.004	0.653 ± 0.002	0.637 ± 0.004	0.643 ± 0.012	0.731 ± 0.008
	35M	SWE	0.617 ± 0.002	0.632 ± 0.009	0.235 ± 0.004	0.669 ± 0.003	0.627 ± 0.002	0.693 ± 0.024	0.690 ± 0.009
		Avg	0.617 ± 0.001	0.628 ± 0.008	0.237 ± 0.002	0.667 ± 0.001	0.648 ± 0.001	0.666 ± 0.024	0.735 ± 0.005
	150M	SWE	0.623 ± 0.002	0.633 ± 0.006	0.248 ± 0.003	0.677 ± 0.003	0.633 ± 0.005	0.663 ± 0.021	0.681 ± 0.011
		Avg	0.624 ± 0.002	0.635 ± 0.006	0.251 ± 0.004	0.679 ± 0.003	**0.661 ± 0.004**	0.676 ± 0.023	**0.754 ± 0.008**
	650M	SWE	**0.629 ± 0.002**	**0.668 ± 0.001**	**0.259 ± 0.004**	**0.685 ± 0.002**	0.630 ± 0.008	**0.810 ± 0.004**	0.643 ± 0.023
		Avg	0.626 ± 0.001	0.650 ± 0.004	0.251 ± 0.002	0.684 ± 0.002	0.651 ± 0.003	0.732 ± 0.030	0.732 ± 0.008
Test	8M	SWE	0.637 ± 0.004	0.638 ± 0.013	0.274 ± 0.008	0.688 ± 0.005	0.636 ± 0.007	0.642 ± 0.030	0.632 ± 0.027
		Avg	0.639 ± 0.000	0.637 ± 0.005	0.279 ± 0.001	0.690 ± 0.000	0.641 ± 0.004	0.633 ± 0.013	**0.645 ± 0.013**
	35M	SWE	0.646 ± 0.003	0.661 ± 0.010	0.294 ± 0.004	0.696 ± 0.005	0.635 ± 0.011	0.690 ± 0.035	0.602 ± 0.040
		Avg	0.648 ± 0.000	0.651 ± 0.010	0.297 ± 0.001	0.699 ± 0.000	0.646 ± 0.008	0.658 ± 0.029	0.639 ± 0.028
	150M	SWE	0.649 ± 0.002	0.664 ± 0.007	0.299 ± 0.005	0.703 ± 0.002	0.636 ± 0.005	0.696 ± 0.020	0.601 ± 0.020
		Avg	0.651 ± 0.001	0.654 ± 0.009	0.303 ± 0.003	0.708 ± 0.002	**0.649 ± 0.006**	0.659 ± 0.025	0.643 ± 0.023
	650M	SWE	0.650 ± 0.005	**0.683 ± 0.007**	0.307 ± 0.007	0.705 ± 0.005	0.625 ± 0.012	**0.754 ± 0.033**	0.545 ± 0.042
		Avg	**0.657 ± 0.002**	0.670 ± 0.006	**0.315 ± 0.003**	**0.717 ± 0.001**	0.646 ± 0.007	0.696 ± 0.022	0.618 ± 0.024

Table S1: PPI validation and test results on the "gold standard" dataset by [S3] for the SWE and average pooling methods across four different ESM-2 PLM backbones. Following [S7], for a comprehensive evaluation, we report the performance using seven different metrics, including accuracy, F1-score, Matthews correlation coefficient (MCC), AUPR, precision, recall, and specificity, with mean and standard deviation across five different random seeds. Underlined numbers indicate the best aggregation performer in each metric for each phase (validation/test) and each PLM. Bold numbers indicate the best performer in each metric for each phase (validation/test).

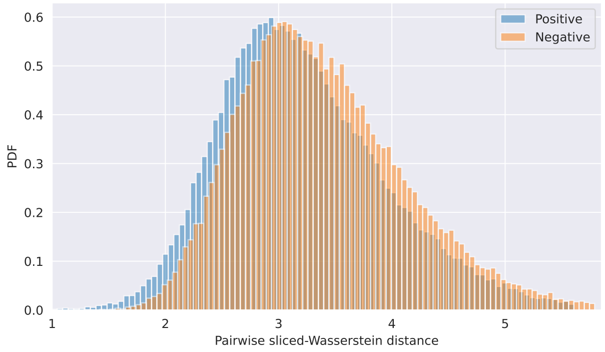

Figure S5: Histograms of sliced-Wasserstein distance between embeddings of interacting and non-interacting proteins at the output space of a pre-trained ESM-2 PLM with 650M parameters. The SW distance is calculated using a trained SWE pooling module with $m = 1024$ reference points and $L = 1024$ slices.

E.1 Visualizing the Distribution of the Learned SWE Representations

One of the desirable properties of the proposed embeddings is that the sliced-Wasserstein distance of the distributions underlying the token-level embeddings of two given protein sequences can be approximated by the average distance of their Monge couplings to the reference across different slices [S6]. In particular, for two proteins $\overline{\mathbf{p}}$ and $\underline{\mathbf{p}}$ with Monge coupling matrices $\overline{\mathbf{Z}} = [\overline{\mathbf{z}}^1, \ldots, \overline{\mathbf{z}}^L]^T \in \mathbb{R}^{L\times m}$ and $\underline{\mathbf{Z}} = [\underline{\mathbf{z}}^1, \ldots, \underline{\mathbf{z}}^L]^T \in \mathbb{R}^{L\times m}$, respectively, we can approximate their pairwise sliced-Wasserstein distance as

$$\mathcal{SW}_2(\overline{\mathbf{p}}, \underline{\mathbf{p}}) \approx \left(\frac{1}{L}\sum_{l=1}^{L}\left\|\overline{\mathbf{z}}^l - \underline{\mathbf{z}}^l\right\|_2^2\right)^{\frac{1}{2}}, \tag{S1}$$

where, with a slight abuse of notation, we use $\mathcal{SW}_2(\overline{\mathbf{p}}, \underline{\mathbf{p}})$ to denote the sliced-Wasserstein distance between token-level representations of $\overline{\mathbf{p}}$ and $\underline{\mathbf{p}}$ at the output of the PLM backbone.

The approximation in (S1) allows us to visualize the pairwise distance of interacting and non-interacting proteins in the embedding space. Figure S5 shows the distributions of (approximate) SW distances between proteins in the training dataset separated by whether or not they interact, where the embeddings are generated by the 650M-parameter ESM-2 backbone and a trained SWE aggregation module with $m = 1024$ reference points and $L = 1024$ slices. As the figure demonstrates, there is a separation between the two histograms, with interacting proteins landing closer to each other in the embedding space from a sliced-Wasserstein distance point of view as compared to non-interacting pairs of proteins. Nevertheless, the difference observed between the interacting and non-interacting histograms does not rise to the level of statistical significance. More work is required to enable the interpretability of the learned SWE representations in PPI tasks and beyond.

References

[S1] Yikun Bai, Ivan Vladimir Medri, Rocio Diaz Martin, Rana Shahroz, and Soheil Kolouri. Linear optimal partial transport embedding. In *International Conference on Machine Learning*, pages 1492–1520. PMLR, 2023.

[S2] Yikun Bai, Bernhard Schmitzer, Matthew Thorpe, and Soheil Kolouri. Sliced optimal partial transport. In *Proceedings of the IEEE/CVF Conference on Computer Vision and Pattern Recognition*, pages 13681–13690, 2023.

[S3] Judith Bernett, David B. Blumenthal, and Markus List. Cracking the black box of deep sequence-based protein-protein interaction prediction. *bioRxiv*, 2023.

[S4] Diederik P Kingma and Jimmy Ba. Adam: A method for stochastic optimization. *arXiv preprint arXiv:1412.6980*, 2014.

[S5] Ilya Loshchilov and Frank Hutter. Decoupled weight decay regularization. In *International Conference on Learning Representations*, 2019.

[S6] Navid NaderiAlizadeh, Joseph F Comer, Reed Andrews, Heiko Hoffmann, and Soheil Kolouri. Pooling by sliced-Wasserstein embedding. *Advances in Neural Information Processing Systems*, 34:3389–3400, 2021.

[S7] Samuel Sledzieski, Meghana Kshirsagar, Minkyung Baek, Rahul Dodhia, Juan Lavista Ferres, and Bonnie Berger. Democratizing protein language models with parameter-efficient fine-tuning. *Proceedings of the National Academy of Sciences*, 121(26):e2405840121, 2024.
